# Haplotype‐based genotyping‐by‐sequencing in oat genome research

**DOI:** 10.1111/pbi.12888

**Published:** 2018-03-25

**Authors:** Wubishet A. Bekele, Charlene P. Wight, Shiaoman Chao, Catherine J. Howarth, Nicholas A. Tinker

**Affiliations:** ^1^ Ottawa Research and Development Centre Agriculture and Agri‐Food Canada Ottawa ON Canada; ^2^ USDA‐ARS Cereal Crops Research Unit Red River Valley Agricultural Research Center Fargo ND USA; ^3^ Institute of Biological, Environmental and Rural Sciences Aberystwyth University Aberystwyth UK

**Keywords:** haplotype, genotyping‐by‐sequencing, *Avena sativa*, genomics‐assisted breeding

## Abstract

In a de novo genotyping‐by‐sequencing (GBS) analysis of short, 64‐base tag‐level haplotypes in 4657 accessions of cultivated oat, we discovered 164741 tag‐level (TL) genetic variants containing 241224 SNPs. From this, the marker density of an oat consensus map was increased by the addition of more than 70000 loci. The mapped TL genotypes of a 635‐line diversity panel were used to infer chromosome‐level (CL) haplotype maps. These maps revealed differences in the number and size of haplotype blocks, as well as differences in haplotype diversity between chromosomes and subsets of the diversity panel. We then explored potential benefits of SNP vs. TL vs. CL GBS variants for mapping, high‐resolution genome analysis and genomic selection in oats. A combined genome‐wide association study (GWAS) of heading date from multiple locations using both TL haplotypes and individual SNP markers identified 184 significant associations. A comparative GWAS using TL haplotypes, CL haplotype blocks and their combinations demonstrated the superiority of using TL haplotype markers. Using a principal component‐based genome‐wide scan, genomic regions containing signatures of selection were identified. These regions may contain genes that are responsible for the local adaptation of oats to Northern American conditions. Genomic selection for heading date using TL haplotypes or SNP markers gave comparable and promising prediction accuracies of up to *r* = 0.74. Genomic selection carried out in an independent calibration and test population for heading date gave promising prediction accuracies that ranged between *r* = 0.42 and 0.67. In conclusion, TL haplotype GBS‐derived markers facilitate genome analysis and genomic selection in oat.

## Introduction

Globally, cultivated oat (*Avena sativa*) is the sixth most important cereal crop. It is grown in temperate regions for grain, and in subtropical regions for forage. Both grain and forage are used for feed, while the grain provides a nutritional human food with documented health benefits (Katz, 2001). To meet new challenges in oat variety development, many breeders are investigating the use of tools for molecular breeding. However, the necessary genomic tools have been difficult to develop in oat because of its large (12.5 GB), repetitive allopolyploid genome (Yan *et al*., 2016b), which has not yet been fully sequenced.

Technological advances in DNA sequencing are revolutionizing biological sciences. Genotyping‐by‐sequencing (GBS) and similar methods (Elshire *et al*., 2011; Truong *et al*., 2012) are applications of this technology. They provide economically, high‐throughput genotyping, which has been applied in crops such as wheat, Miscanthus and oat without the need for a complete reference genome (Huang *et al*., 2014; Lu *et al*., 2013; Poland *et al*., 2012). Markers based on GBS have been used in genome‐wide association studies (GWAS) and in genomic selection (Morris *et al*., 2013; Poland *et al*., 2012).

Genetic linkage mapping and diversity studies conducted using array‐based SNPs, and first‐generation GBS markers have helped us to gain insight into the complex oat genome (Chaffin *et al*., 2016; Esvelt Klos *et al*., 2016; Huang *et al*., 2014). However, the limited number of GBS markers and lack of a standardized nomenclature encouraged us to develop improved methods for GBS analysis, which resulted in the development of computer software called ‘Haplotag’ (Tinker *et al*., 2016). This software provides an efficient analysis tool for oat and other complex genomes for which no reference sequence is available. Haplotag employs population‐level model filtering to identify sets of tag‐level (TL) haplotypes that show diploid segregation (Tinker *et al*., 2016). What makes Haplotag unique is its output of a set of genotype inferences for TL haplotypes, also referred to as ‘Haplotag Loci’. As these TL haplotypes may contain multiple SNPs, Haplotag will also produce an alternate set of genotypes based on the underlying SNP calls (GBS‐SNPs). Furthermore, Haplotag operates in either a production mode or a discovery mode. The discovery mode involves *de novo* clustering and genotype calling, whereas the production mode calls genotypes from a predefined set of haplotypes (Tinker *et al*., 2016).

Haplotype‐based genetic analyses have been used in human, animal and plant genetics research. Such haplotypes are normally inferred either from a genome sequence, or through linkage or association analysis. Hereafter, we refer to these as chromosome‐level (CL) haplotypes, to differentiate them from the TL haplotypes that are inferred directly from GBS tags by Haplotag. In comparison with using individual SNPs, haplotype‐based analysis can reduce false discovery rates because it performs fewer association tests (Hamblin and Jannink, 2011). Performing fewer association tests requires less computational time, but more importantly, using fewer tests that still cover the same independent variable space can provide increased statistical power (Rafalski, 2002).

Simulation studies that compared genomic selection using CL haplotypes vs. genomic selection using SNPs showed that selection accuracies of CL haplotypes were lower than those based on SNP markers (Jannink *et al*., 2010). However, recent empirical comparisons using higher marker densities revealed that CL haplotype‐based genomic selection gave slightly higher genomic prediction accuracy than did individual SNPs (Cuyabano *et al*., 2014, 2015; Edriss *et al*., 2013). One of the problems associated with CL haplotypes is the possibility that the predicted haplotype data might be compromised by errors in map construction. In contrast, the short TL haplotypes derived from Haplotag analysis extend over distances of only 64 bp, and their accuracy is not affected by map errors. We are not aware of any work to date that has evaluated GWAS or genomic selection based on TL haplotypes.

The primary goal of this study was to evaluate the suitability of GBS‐derived TL haplotype for breeding and genomics research using empirical data from cultivated hexaploid oat. The model phenotypic‐trait heading date was used for analysis because of its importance for local adaptation, and the availability of several published genetic mapping studies to compare with. The Haplotag GBS pipeline was applied to more than 4600 cultivated oat lines, and the marker data were used in four studies: (i) genetic linkage mapping (TL haplotype vs. GBS‐SNPs), (ii) population genomics and haplotype mapping of elite North American lines, (iii) GWAS analysis of heading date (GBS‐SNPs vs. TL haplotype vs. CL haplotypes) and (iv) genomic selection using TL haplotype vs. GBS‐SNPs.

## Results

### Oat tag‐level haplotype markers

Using the *de novo* discovery mode of Haplotag, we called 164741 TL haplotype loci with 353130 TL alleles and 241224 GBS‐SNP markers from 4657 cultivated oat lines. These lines consisted of mapping population lines, breeding lines and germplasm material. To our knowledge, this is the largest number of cultivated oat lines that have been addressed in a single analysis. The complete data matrices and the supporting Haplotag input files can be downloaded in a set of annotated text files, while the complete set of genotype calls and map locations of the markers (see below) have been fully integrated into the T3/oat platform (http://triticeaetoolbox.org/oat/genotyping) (Saied *et al*., 2016). The marker data were filtered from these matrices based on the appropriate taxa set and population‐level parameters for each respective analysis.

### Updated oat consensus map

The updated oat consensus map (Appendix S1) contains a comprehensive set of 99878 mapped markers. This number includes the 74461 new Haplotag‐derived markers, and the complete set of markers that were reported by Chaffin *et al*. (2016). A total of 19074 legacy GBS loci can be recognized by the ‘avgbs’ prefix followed by a number with no decimals. The new Haplotag loci have either a single decimal (for TL haplotype loci) or two decimals (for SNP loci, where the second decimal identifies the SNP position). The positions of corresponding Haplotag markers (TL vs. SNP) were identical or within a few cM of each other. As illustrated in Figure S1, there were up to 861 markers within each 1 cM bin of the GBS‐SNP map, and up to 666 markers within each bin of the TL haplotype map. Overall, marker placement using the two systems gave similar results, as revealed by the high correlation (*r* = 0.99) between the two 1 cM bin maps. The average number of markers per bin was 10.6 for the TL haplotype map and 14.9 for the GBS‐SNP map.

Because the Haplotag pipeline groups and names loci based on clusters of similar tags, it was not possible to cross‐reference all Haplotag SNP and legacy GBS loci. However, we preserved the legacy nomenclature of 6239 Haplotag SNPs belonging to tags that clustered into a single pair of haplotypes containing only one SNP. The positions of these cross‐referenced loci are shown in the second page of Appendix S1. Of these, 187 (2.9%) mapped to different groups, and 290 (4.6%) mapped to positions separated by more than 10 cM. As the algorithms used to place markers on the framework were identical, these discrepancies are most likely caused by the addition of three new mapping populations that were not included by Chaffin *et al*. (2016). These populations were included to expand the genotype diversity for Haplotag allele discovery, and full reports of *de novo* map construction and phenotypic analysis in these populations may be topics of future work.

### A haplotype map of oat

Mapped TL haplotype loci and GBS‐SNPs were used to investigate CL haplotype structure in the oat diversity panel (*n* = 635). Across all lines, the linkage disequilibrium (LD)‐based haplotype detection method (Gabriel *et al*., 2002) identified 754 and 3495 CL haplotype blocks using TL loci or GBS‐SNPs, respectively. The reduced number of haplotypes based on TL markers is due to the compression of data for each TL locus into a pair of major and minor alleles. Within subpopulations, 1793 or 1319 CL haplotype blocks were identified based on the TL loci found in the spring (*n* = 497) and southern (*n* = 123) sets of germplasm, respectively. The CL haplotype blocks in the full set covered 246 cM, while those in the southern set covered 573.9 cM and those in the spring set covered 521.2 cM. The genome‐wide average haplotype block sizes were 0.31, 0.29 and 0.43 cM (Table 2) for the full, spring and southern subpopulations, respectively. The comparison between subpopulations using GBS‐SNP‐derived haplotype blocks showed similar trends.

**Table 1:** Summary of marker placement

Linkage group	Number of Haplotag‐derived markers	Tag‐level haplotype markers				GBS‐SNP markers			
		Number of markers	Minimum position (cM)	Maximum Position (cM)	Size (cM)	Number of markers	Minimum position (cM)	Maximum Position (cM)	Size (cM)
Mrg01	4909	2077	−11.80	142.30	154.10	2832	−11.80	142.30	154.10
Mrg02	4122	1694	−1.90	118.50	120.40	2428	−1.90	118.50	120.40
Mrg03	4816	2040	−0.30	162.00	162.30	2776	−0.30	162.00	162.30
Mrg04	1859	777	−17.30	79.70	97.00	1082	−17.30	79.70	97.00
Mrg05	2986	1212	0.60	175.30	174.70	1774	−10.60	175.30	185.90
Mrg06	3265	1342	−1.90	149.20	151.10	1923	−1.90	149.20	151.10
Mrg08	3370	1383	0.00	203.70	203.70	1987	0.00	203.70	203.70
Mrg09	3716	1640	−8.60	140.40	149.00	2076	−8.60	140.40	149.00
Mrg11	3866	1663	−14.40	109.60	124.00	2203	−14.40	109.60	124.00
Mrg12	3880	1562	4.20	125.50	121.30	2318	4.20	125.50	121.30
Mrg13	2890	1266	1.20	127.30	126.10	1624	1.20	127.30	126.10
Mrg15	3710	1611	−7.10	93.10	100.20	2099	−7.10	93.10	100.20
Mrg17	4854	2075	2.40	115.60	113.20	2779	2.40	115.60	113.20
Mrg18	3194	1302	−1.90	120.60	122.50	1892	−1.90	120.60	122.50
Mrg19	2025	860	−17.20	93.20	110.40	1165	−17.20	92.20	109.40
Mrg20	4574	1754	15.80	261.00	245.20	2820	15.80	261.00	245.20
Mrg21	5024	2050	−4.10	215.80	219.90	2974	−4.10	216.00	220.10
Mrg23	2741	1113	8.10	124.90	116.80	1628	8.10	124.90	116.80
Mrg24	3244	1342	−0.50	95.30	95.80	1902	−0.50	95.30	95.80
Mrg28	3518	1485	−2.80	104.40	107.20	2033	−2.80	104.40	107.20
Mrg33	1898	747	−7.90	131.40	139.30	1151	−7.90	131.40	139.30

**Table 2:** Summary of haplotype blocks

Linkage group	Full diversity panel haplotype blocks				Spring set haplotype blocks				Southern set haplotype blocks			
	Number	Minimum size (cM)	Maximum size (cM)	Mean size (cM)	Number	Minimum size (cM)	Maximum size (cM)	Mean size (cM)	Number	Minimum size (cM)	Maximum size (cM)	Mean size (cM)
Mrg01	37	0	1.2	0.21	134	0	3.7	0.15	27	0	1.29	0.13
Mrg02	97	0	8.7	0.52	78	0	11.5	0.34	40	0	7.9	0.56
Mrg03	17	0	1.2	0.28	131	0	4.3	0.16	23	0	0.8	0.14
Mrg04	14	0	2.5	0.48	41	0	9.3	0.3	13	0	2.5	0.43
Mrg05	28	0	1.2	0.3	77	0	16.5	0.46	12	0	1.29	0.21
Mrg06	40	0	5.5	0.21	82	0	8.5	0.35	24	0	1.7	0.14
Mrg08	21	0	4.6	0.33	79	0	3.09	0.34	16	0	6.4	0.81
Mrg09	28	0	2.5	0.27	103	0	3.2	0.25	27	0	2	0.31
Mrg11	49	0	1.2	0.18	105	0	2.9	0.17	42	0	6.29	0.37
Mrg12	34	0	4.2	0.39	81	0	6.59	0.29	38	0	6.5	0.49
Mrg13	31	0	0.8	0.06	82	0	2.59	0.18	33	0	2.69	0.39
Mrg15	66	0	2	0.19	72	0	3.4	0.23	35	0	2.79	0.52
Mrg17	32	0	1.6	0.22	159	0	3.09	0.18	30	0	2	0.22
Mrg18	46	0	2.1	0.27	75	0	2.9	0.27	14	0	1.39	0.37
Mrg19	18	0	1.1	0.18	50	0	4.4	0.27	15	0	6.09	0.67
Mrg20	29	0	1.4	0.18	116	0	13	0.62	20	0	6.8	0.79
Mrg21	31	0	1.7	0.19	107	0	5.4	0.29	26	0	20.5	1.09
Mrg23	19	0	7.4	0.57	37	0	5.69	0.55	15	0	4.3	0.89
Mrg24	37	0	2	0.32	79	0	5.19	0.24	21	0	2.3	0.36
Mrg28	66	0	15.2	0.66	59	0	7.9	0.34	26	0	8.2	0.69
Mrg33	14	0	5.7	0.59	46	0	6	0.57	7	0	1.4	0.53

In the analysis of the full germplasm set, linkage groups Mrg02 and Mrg28 contained a large number of haplotype blocks, as well as some of the longest haplotype blocks (Table 2). The maximum number of TL haplotype markers per block was 49 markers, covering 4.2 cM (32.6–36.8 cM) on Mrg28. The second largest number of markers was also on Mrg28, at position 43.8 cM. This haplotype block consisted of 19 markers with a 0 cM block size. The spring and southern sets showed differences in frequency and size of chromosome‐level haplotypes (Table 2).

Haplotype diversity in the full set and the subpopulations showed differences between subpopulations and genomic regions (Figures  S2 and S3). In the full set, the lowest mean chromosome haplotype diversity was on Mrg13, followed by Mrg28 (Figure S2). However, there were fewer haplotype blocks detected on Mrg13 compared with Mrg28. The highest mean chromosome haplotype diversity was on Mrg05, followed by Mrg02. Mrg02 and Mrg28 contained the two largest numbers of haplotype blocks per chromosome, but showed contrasting mean haplotype diversity (Figure S3a).

### Genome‐wide association using Haplotag‐derived markers and chromosome‐level haplotypes

We conducted two sets of GWAS comparisons for heading date using the CORE diversity panel (*n* = 635) heading data from 16 location‐years. The first set compared two types of Haplotag‐derived markers (TL haplotype vs. GBS‐SNP) and was performed separately for each environment using the full diversity panel, as well as the spring and southern subsets. The second set compared Haplotag‐derived markers vs. CL haplotypes using BLUP values across environments using the full diversity panel (Table S1).

The first GWAS identified 184 significant associations across the two marker systems after Bonferroni correction (Appendix S3). These analyses were conducted using 12890 TL haplotype (MAF ≥0.05) and 17694 GBS‐SNP (MAF ≥0.05) markers. These included 115 significant TL associations exceeding the 5% Bonferroni threshold (−log_10_ *P* ≥5.41) vs. only 69 GBS‐SNP associations (−log_10_ *P* ≥5.55). GWAS conducted in the spring and southern sets showed the same trend, although the differences were smaller. The two chromosome representations with the most significant associations were Mrg02 and Mrg12. On Mrg02, there were 20 loci at position 34 cM associated with heading dates from eight field trials. On Mrg12, there were 23 TL haplotype markers at positions 40–42 cM associated with heading dates from seven locations (Appendix S3).

**Figure 1:**
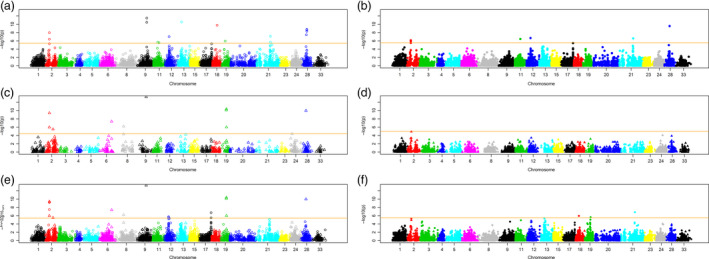
Manhattan plots for TL haplotype (left)‐ and GBS‐SNP (right)‐based genome‐wide association scans. The 21 chromosome representations from the oat consensus map are shown on the horizontal axis, and −log10(*P*) values of association tests at each marker are shown on the vertical axis. The horizontal orange lines show the Bonferroni threshold (*P* = 0.05) for each respective marker system. The upper plots show the GWAS result using each of the two marker systems alone (a, b), followed by GWAS using only the CL haplotypes (c, d), and the lower plots show GWAS results using the union of CL haplotype and original markers (e, f), excluding the markers that are components of the respective CL haplotypes.

We then compared GWAS scans based on Haplotag‐derived markers vs. those based on CL haplotypes. Parallel GWASs were performed on the same BLUP‐based phenotype data using the two Haplotag‐derived marker systems, CL haplotypes derived independently from each of these two systems, and CL haplotypes combined with markers that were not included in their respective CL haplotypes. All systems except the GBS‐SNP‐derived CL haplotypes detected significant associations after Bonferroni threshold (*P* = 0.05) (Figure 1; noting that each marker system has a different threshold, depending on the number of markers). In general, the patterns of association were similar using any of the systems, but most of the marker systems detected additional unique genomic regions. For example, the significant association on Mrg09 at position 78 was only detected by three TL‐based methods. The significant hits on Mrg06 and Mrg08 were only detected by the methods that included CL haplotypes based on TL markers, and the effects on Mrg11 were only detected by the individual TL and SNP marker systems. Overall, the TL analyses (Figure 1a,c,e) detected the largest numbers of unique regions of association. Despite the low proportion of CL haplotype markers (10.9%) in the combined TL‐CL haplotype analysis, most (10/17) of the significant GWAS effects were based on the CL haplotypes within this analysis. These hits were mapped across eight chromosomes, with six significant associations being found on Mrg02. Five of these associations were at the 34 cM position (Appendix S3).

### Genome scan for loci related to local adaptation in oats

**Figure 2:**
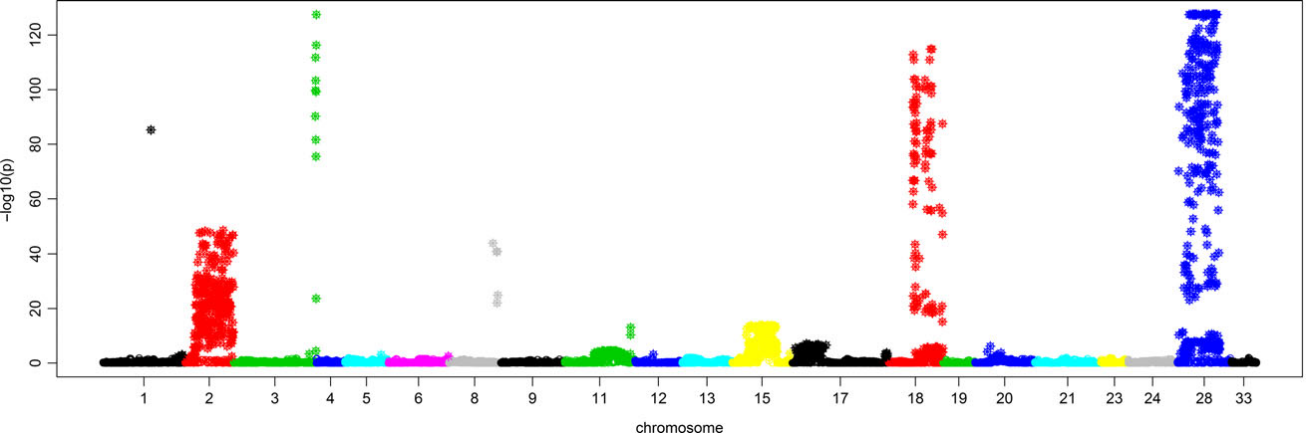
PCA‐based genome‐wide scan for selection. The Manhattan plot shows −log10(*P*) on the vertical axis. Significant *P*‐values below a threshold false discovery rate of (∞ = 0.05) are indicated by stars.

We used TL haplotype markers from the CORE diversity panel (*n* = 635) and applied a PCA‐based outlier detection method called ‘pcadapt’. The first step in this analysis identified the first nine principal components. *K* = 9 was selected because it appeared as the highest point before the beginning of a plateau at approximately *K* = 10 (Figure S4). Regression of the markers on the first nine principal components identified 1610 TL haplotype markers at the false discovery rate (*q*‐value) threshold (∞ ≤ 0.05). These loci were distributed across thirteen chromosome representations (Figure 2), with 98% on six chromosome representations (Mrg02, Mrg28, Mrg15, Mrg11, Mrg17 and Mrg18) (Table S2). These significant markers were distributed in 97 1 cM‐bins, representing 6.7% of the oat consensus map.

### Haplotag‐derived markers for genomic selection in oats

**Figure 3:**
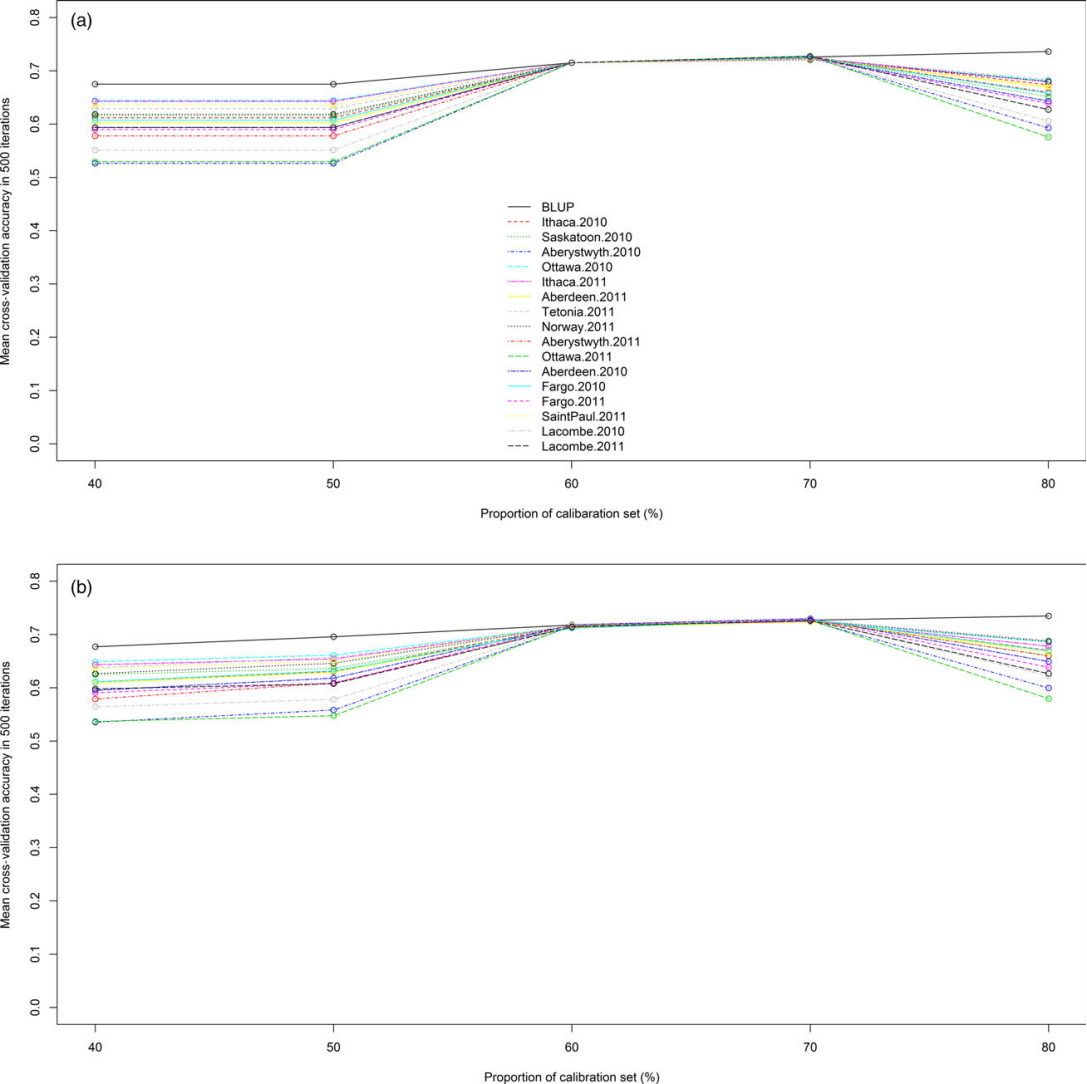
Cross‐validation accuracy of the CORE diversity panel (*n* = 635) using TL haplotype (a) and GBS‐SNP markers (b). Heading date data from 16 location‐by‐year combinations and the line BLUP values are represented by different colours and line patterns. The *x*‐axis shows the calibration set sizes, and the *y*‐axis represents mean correlations of predicted phenotypic values to observed heading date.

We applied genomic prediction with an RR‐BLUP mixed model for heading date using TL haplotype loci and GBS‐SNPs. The two marker systems were compared in a cross‐validation analysis of the diversity set (*n* = 635). The first cross‐validation comparison used random calibration sets comprising 40% to 80% of the diversity panel, with the remaining unselected lines used to make up the test sets. The mean cross‐validation accuracies of the GBS‐SNP and TL haplotype markers showed no statistically significant differences, and both reached a plateau at a calibration set size of 60% (Figure 3). Prediction accuracy declined at 80% calibration set in all phenotypic values except the BLUP ones.

**Figure 4:**
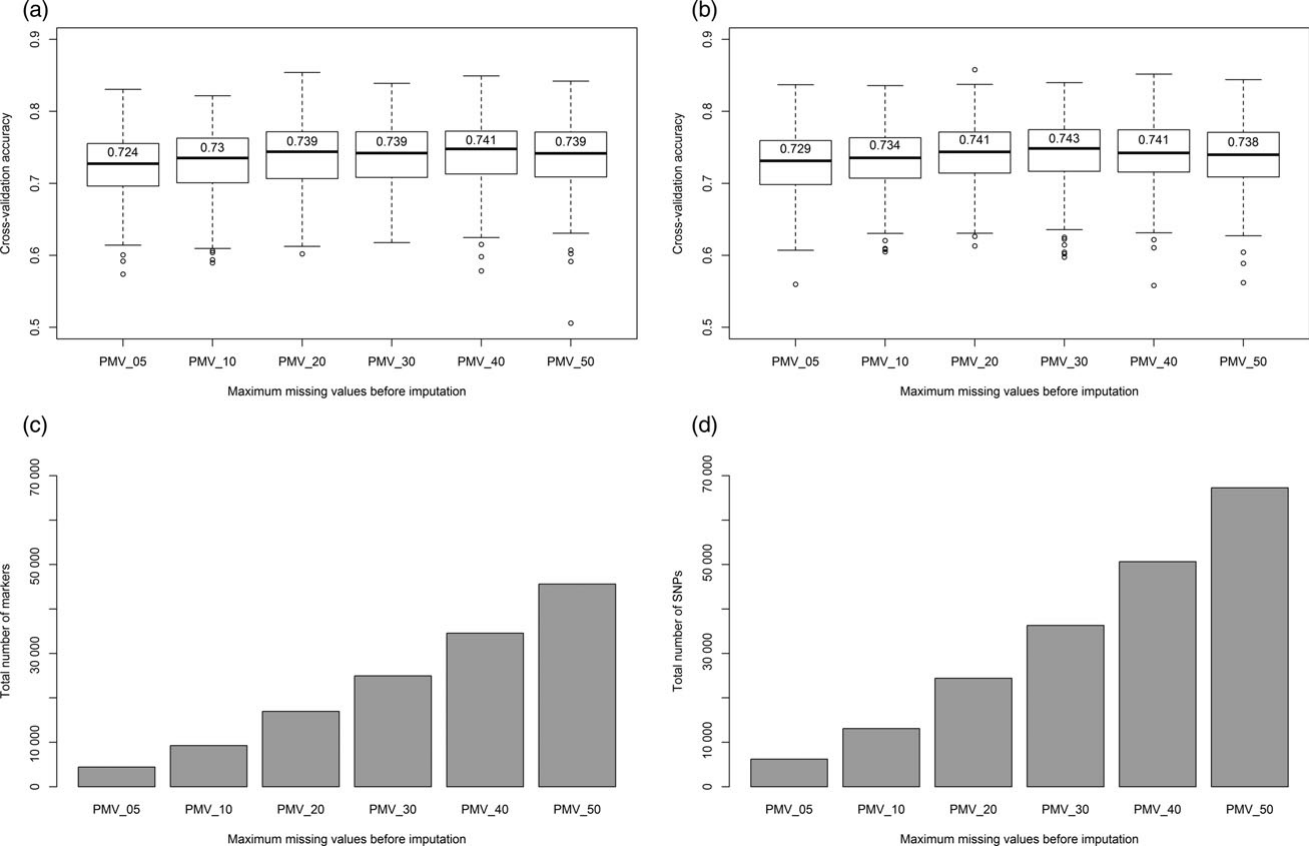
Prediction accuracy of the heading date BLUP using six levels of maximum percentage missing values (PMV) before imputation. Predictions using TL haplotype markers are shown on the left (a, c), while predictions with SNP markers are shown on the right (b, d). The bar graphs at the bottom (c, d) show the total number of markers used for the genomic selection model. The top boxplots (a, b) show the results of 500 iteration cross‐validation accuracies. The values on top of the median line show mean cross‐validation accuracies.

Marker imputation on data sets ranging from 5% to 50% missing values increased the number of markers by more than tenfold (Figure 4). However, this increase in marker number resulted in a <2% increase in genomic selection accuracy. Furthermore, the differences in mean accuracies using 20%, 30% or 40% missing markers were not statistically significant.

An independent validation was performed using Home‐test 2010 lines as test set and the diversity set of lines (*n* = 635) as calibration set. Independent predictions were computed using heading dates from 16 environments as calibration set phenotypes. The prediction accuracies were calculated as the correlation between the predicted heading date values with the observed phenotype from Home‐test 2010 at Prince Edward Island, Canada (Figure S5), which ranged from 0.42 to 0.67. The calibration set phenotype from Ithaca 2010 had the highest independent validation accuracy (*r* = 0.67). The genomic selection model using GBS‐SNPs gave slightly higher accuracy than the model using TL haplotype markers.

## Discussion

### Haplotag‐enabled high‐throughput genotyping and an updated consensus map in oats

We used the Haplotag software to analyse GBS data from 4657 cultivated oat lines and generated more than 400000 TL haplotype and GBS‐SNP markers. This greatly increased the number of markers available for genomics‐assisted breeding and population genomics studies in cultivated oat. Genomic tools in the form of fixed arrays or common sets of GBS markers have been deployed by many crop breeding communities, including oat (Huang *et al*., 2014; Tinker *et al*., 2009, 2014), wheat (Jordan *et al*., 2015; Wang *et al*., 2014), maize (Glaubitz *et al*., 2014) and sorghum (Morris *et al*., 2013). These publicly available genotype data have been used by research groups from around the world (Boyles *et al*., 2016; Zhang *et al*., 2015). Oat researchers wishing to build on our results using a common marker set can use the publicly available Haplotag software in production mode, together with the nomenclature files that we have provided.

The updated consensus map and the accompanying diversity data enabled us to conduct comparative GWAS and to infer the first haplotype map of oat. This map was also used recently to develop a chromosome‐specific analysis of ancestral genome contributions in wild and cultivated oat (Yan *et al*., 2016a). Several *Avena* genome assembly projects, especially the sequencing of the cultivated hexaploid oat genome, will benefit from this map, as was the case in the barley and rice map‐based reference genome assemblies (IBGSC, 2012; IRGS, 2005).

Certain genomic regions on Mrg28, Mrg02, Mrg12, Mrg15, Mrg24, Mrg21 and Mrg11 contained more than 250 markers per bin (Figure S1). Recombination rate is influenced by the chromosome position of a marker, centromeres, chromatin structure, nucleotide content and any major structural rearrangements. Recombination hot spots in maize are associated with reduced genetic load (Rodgers‐Melnick *et al*., 2015). Conversely, recombination cold regions could be due to the clustering of adaptive loci (Yeaman, 2013). Many of these recombination‐suppressed regions in oat may coincide with translocations, or they may represent important QTL hot spots for adaptive traits.

### The first oat haplotype map

Our report of the first oat CL haplotype map provides insight into the haplotype structure of cultivated oat lines from North America. We observed differences in the number and size of haplotype blocks between spring and southern lines. As haplotype structure is related to LD, these differences are consistent with differences in LD decay observed by Esvelt Klos *et al*. (2016) and may indicate footprints of adaptive QTL. These differences could have resulted from natural or artificial selection during breeding for different agro‐climatic conditions (e.g., northern vs. southern or spring vs. winter production). Such conditions can influence patterns of genetic variation in elite oat lines (Esvelt Klos *et al*., 2016; Fu *et al*., 2003; Grau Nersting *et al*., 2006; Montilla‐Bascón *et al*., 2013). For example, in the full set analysis, Mrg02 had the largest number of haplotype blocks, one of which is the fourth‐longest haplotype block (88.7–97.4 cM), which is close to loci affecting heading date (De Koeyer *et al*., 2004; Locatelli *et al*., 2006) and rust resistance (Esvelt Klos *et al*., 2017; Wight *et al*., 2004). On the other hand, the spring set haplotype analysis identified the third‐longest haplotype block spanning 11.5 cM (42–53.5 cM) on Mrg02. Esvelt Klos *et al*. (2016) reported that Mrg02 showed a slower LD decay rate in the spring population compared to the southern/winter set prior to correcting for population structure and kinship. The second longest haplotype block in the spring set analysis is on Mrg20, spanning 122.8–135.8 cM, which is one of genomic regions associated with crown rust resistance in oat (Esvelt Klos *et al*., 2017). In the southern set, Mrg21 harbours the biggest haplotype block (20.5 cM), and this is close to the oat vernalization locus *Vrn2* (Nava *et al*., 2012). The regions homeologous to *Vrn2* on Mrg20 and Mrg12 (Nava *et al*., 2012) also contain several haplotype blocks, including the third‐longest block on Mrg12 (48.7–51.7 cM). These genomic regions on Mrg20 and 21 were detected by a recent GWAS that investigated frost tolerance in European oat lines (Tumino *et al*., 2016).

Breeding or artificial selection can also change the frequency of selected haplotypes (Yonemaru *et al*., 2012). Haplotype diversity is the function of the number of alleles/haplotypes, and their frequency in a population. Genome‐wide CL haplotype diversity analysis identified chromosomes that show differences between the spring and southern sets (Figure S3). The southern set showed higher mean CL haplotype diversity compared to the spring set, except on Mrg04, 05, 08, 18, 23 and 24—regions that harbour vernalization and heading date QTL (Holland *et al*., 2002; Tumino *et al*., 2016). Our hypothesis is that these regions contain specific daylength‐ and vernalization‐related alleles that are highly selected within the southern germplasm. There are other examples of breeding‐induced reductions in haplotype diversity, such as the low haplotype diversity surrounding the rice heading date gene (Yonemaru *et al*., 2012). Similarly, selective sweeps and differential selection in wheat and sorghum breeding programmes have resulted in regions of reduced haplotype diversity associated with the adaptation of these crops to different growth habits or temperate agro‐climatic conditions (Cavanagh *et al*., 2013; Mace *et al*., 2013; Morris *et al*., 2013; Thurber *et al*., 2013).

### Haplotype‐based genome‐wide association mapping

In principle, haplotype‐based GWAS has higher statistical power than SNP‐based GWAS because of reduced dimensions or multiple testing, but, in practice, other factors can affect this result. We found that Haplotag‐derived markers effectively substituted for array‐based markers in identifying the major associations on Mrg02 and Mrg12, and that Haplotag‐derived markers identified a large number of additional associations, even after a stringent Bonferroni correction. These included new genomic regions such as that on Mrg09, which was detected only using the three TL methods.

When we compared six different approaches to the analysis of GBS data (GBS‐SNPs, TL haplotype, CL haplotype and their combinations), we found a high degree of similarity, with some differences in the identified genomic regions (Figure 1). Surprisingly, the method based only on SNP‐derived CL haplotypes did not detect any significant associations, while the TL‐derived CL haplotype analysis identified a majority of the common associations. This could be because the TL‐derived CL haplotypes were based on a compression of the TL allele states to a major and minor allele, while the SNP‐derived haplotypes were not. Thus, the SNP‐derived CL haplotypes were more numerous with a greater number of minor alleles, and this may have affected the threshold for error control without an accompanying increase in explanatory power. Overall, TL haplotype‐based analyses identified more significant associations than GBS‐SNP‐based methods. Nevertheless, each system identified unique significant associations. This might be because each QTL region has its own recombination pattern and evolutionary history. Hence, the testing of combinations of multiple marker systems is the most pragmatic approach (Hamblin and Jannink, 2011). Similar empirical GWAS comparisons between haplotype and SNP markers in other crops and animal studies showed mixed results, but the majority of studies reported that haplotype‐based GWAS was superior (Hamblin and Jannink, 2011; Lorenz *et al*., 2010; Visioni *et al*., 2013).

### Adaptation genomics in oats

We identified 1610 markers that are correlated with population structure using a PCA‐based genome‐wide scan (pcadapt). Unlike FST‐based methods, pcadapt does not require the prior grouping of individuals into subgroups (Duforet‐Frebourg *et al*., 2016). This makes it suitable for oat, which has a weak population structure, attributed to an intensive germplasm exchange amongst breeding programmes (Esvelt Klos *et al*., 2016). The two highest–log(*P*) values (Figure 2) were on Mrg18 and Mrg28. FST‐based analysis by Esvelt Klos *et al*. (2016) failed to identify these major translocation regions, and this may further demonstrate the improved sensitivity of the pcadapt method. These two chromosome representations were assigned by Oliver *et al*. (2013) to physical chromosomes suspected to harbour a major reciprocal translocation (7c‐17A). The suspected intergenomic translocation region on Mrg28 (7c‐17A) is associated with winter survival (Wooten *et al*., 2007) and spring growth habit (Jellen and Beard, 2000). The same region has also been associated with traits such as stem rust resistance, plant height and seed oil and beta‐glucan contents (Kianian *et al*., 2000; O'Donoughue *et al*., 1996; Siripoonwiwat *et al*., 1996). The largest number of significant outlier markers was on Mrg02, spanning a region from 27 to 108 cM (Figure 2). This genomic region harbours the two major *HD1* homologous regions, heading date GWAS hits (Esvelt Klos *et al*., 2016) and a cluster of rust resistance genes (Wight *et al*., 2004).

The two major adaptation‐related genomic regions on Mrg02 and Mrg28 could be important signatures of breeding history (Table 3). The co‐occurrence of adaptive QTL, reduced recombination regions, many haplotype blocks and lower haplotype diversity suggests that Mrg28 has been influenced by a selective sweep (Messer and Neher, 2012). Similar results were found in other cereals such as rice and sorghum, where breeding and selection resulted in decreased haplotype or nucleotide diversity around major flowering time‐/maturity‐related genes (Mace *et al*., 2013; Yonemaru *et al*., 2012). The wheat haplotype map (Jordan *et al*., 2015) revealed that wheat breeding favoured adaptive loci and resulted in a selective sweep. The two most structurally rearranged wheat chromosomes (4A and 7B) harboured a large number of loci with extreme FST values. These findings are also in agreement with the role of genomic rearrangements in maintaining clusters of local adaptation‐related loci (Yeaman, 2013). In contrast, the region on Mrg02, which also contains adaptation‐related QTL, has a large number of haplotypes in the spring germplasm, with haplotype diversity that is equal to or above the genome‐wide average (Table 3). This could be explained by the large number of spring lines, and by the creation of new haplotype combinations as a result of breeding for adaptation, but it suggests the absence of a selective sweep. Genome‐wide changes in haplotype diversity during modern rice breeding in Japan include the creation of new haplotypes and increased haplotype diversity (Yonemaru *et al*., 2012). The diversity on Mrg02 in oat could have been driven by the introgression of alleles at a cluster of loci affecting crown rust resistance that was introgressed from *Avena sterilis* (Wight *et al*., 2004). Introgressions from wild relatives might have formed local islands that show high diversity and low recombination. Several *A. sativa* × *A. sterilis* hybrids show meiotic irregularities, distorted segregations and clustering of markers at the same genetic position (McMullen *et al*., 1982; Wight *et al*., 2004). Moreover, the multiple introgressions of genes conferring resistance to different rust races might have increased haplotype diversity. In wheat, the introgression of resistance genes from wild relatives such as *Aegilops tauschii* into the D genome resulted in a large number of outlier loci, haplotypes and high diversity (Jordan *et al*., 2015).

**Table 3:** Summary of two adaptation‐related genomic regions

	Mrg02	Mrg28	Genome‐wide average
Genetic positions of the significant adaptation‐related loci (cM)	27–108	18–44	NA
Significant adaptation‐related loci per 1 cM bin	4.4	13.8	0.4
Consensus map marker density per 1 cM bin (TL haplotype/SNP)	18/25	43/55	11/15
Number of haplotype blocks per 1 cM bin (TL haplotype)	0.97	1.92	0.26
Mean haplotype block size (cM) (all/spring/southern)	0.52/0.36/0.56	0.17/0.29/0.23	0.32/0.25/0.43
Mean haplotype diversity (all/spring/southern)	0.54/0.50/0.58	0.34/0.38/0.45	0.54/0.46/0.51
Significant TL haplotype‐based heading date GWAS hits	23	16	132

### Haplotag‐derived markers for genomic selection

The previous lack of a high‐density marker system limited the application of genomic selection in oat. This was evident from the work of Asoro *et al*. (2011), where the accuracy of genomic selection increased continually up to the limit imposed by the number of available DArT markers. In contrast, cross‐validation using Haplotag‐derived markers reached a plateau of accuracy (Figures 3 and 4), likely because of them having both a higher density and a more even distribution. Similar advantages were reported in wheat (Poland *et al*., 2012).

The maximum mean cross‐validation accuracy obtained was 0.74, using either SNP or TL marker systems. This accuracy is similar to values measured for prediction of heading date in wheat and rice (Isidro *et al*., 2015; Poland *et al*., 2012). Cross‐validation with calibration set to test set proportions of 40% to 60% using the diversity panel (*n* = 635) gave comparable cross‐validation accuracies. Similar results were obtained in maize cross‐validation, especially in traits with high genetic variance (Zhao *et al*., 2012).

Using imputation, we increased the total number of markers from 4423 to 67284. However, this large increase in marker number did not significantly improve the mean cross‐validation accuracy (Figure 4). Similar results were obtained in wheat and other crops using GBS markers (Poland *et al*., 2012). However, future research needs to compare different imputation methods, including map‐based imputation (Rutkoski *et al*., 2013).

The phenotype and genotype data of the CORE diversity set are publically available to the oat breeding community, and breeders can use this resource to predict the performance of lines from their breeding programmes. Figure S5 shows an example of the predictive accuracy of CORE heading date data in the 2010 Ottawa home‐test population. The accuracies obtained for these independent validations were lower than cross‐validation accuracies, which are in agreement with previous independent validation comparisons (Asoro *et al*., 2011; Battenfield *et al*., 2016) and reflect additional variance in environment and/or genotype‐by‐environment interaction between years, as well as potential differences in population parameters. Differential response to environment is a confounding factor in all types of selection; thus, this reduced accuracy of genomic prediction probably reflects a more realistic metric for the selection of stable and predictable performance in practical breeding schemes. The Haplotag build reported here is currently being used for production mode genotype calls of our local breeding germplasm to perform genomic selection. Breeding or research programmes interested in applying a similar approach can download the necessary data from T3/oat.

## Conclusion

The availability of Haplotag‐derived markers in thousands of cultivated oat lines opens the way for genetic analysis, genomic selection, whole‐genome sequencing and other applications of genomics tools in oat. The new set of haplotype loci and alleles can be considered as a high density and highly informative genotyping platform for cultivated oat. We have applied Haplotag‐derived SNP and TL markers in previously studied populations, and validated the superiority of these marker systems. These high‐density markers have enriched the consensus map and improved GWAS. In addition, our comparative study showed that Haplotag‐derived markers can effectively substitute for currently available array‐based SNPs in oat. This high‐density marker system was used to construct the first oat haplotype map and to identify genomic regions that are important for local adaptation. This marker system will be a key tool for the design and implementation of genomics‐based breeding in oats: by generating information about the genetic architecture of traits and/or as a cost‐effective genome‐wide marker for genomic selection.

## Experimental procedures

### Genetic material and phenotype data

A total of 4657 cultivated oat lines from predominantly North American breeding materials were selected for GBS analysis (Appendix S1). Three major sources within this material included the following: (i) ten biparental RIL populations (*n* = 950), of which seven were used in previous consensus map construction (Chaffin *et al*., 2016), (ii) the 635‐line CORE diversity panel consisting of 497 spring and 123 southern lines, (iii) breeding lines from the Ottawa Research and Development Centre (ORDC) and collaborating groups (*n* = 1510). A set of 197 ‘home‐test’ lines from the 2010 Ottawa oat breeding programme were also included to validate prediction accuracy of genomic selection. An additional set of 1248 lines from a public oat genotyping initiative (POGI) were included; however, data from these lines did not contribute to the reported genetic analyses. The POGI lines were included to expand the sampling of TL haplotypes, such that the current map and marker nomenclature would be directly applicable to future studies using this material.

DNA extraction and library preparation for the double digest (PstI‐MspI) GBS system were described in previous work (Huang *et al*., 2014) with minor differences in DNA isolation among the POGI lines.

We used heading date as a model trait for analysis because of its importance in local adaptation and our ability to compare results to those published by Esvelt Klos *et al*. (2016). Heading data from the CORE diversity set from 16 location and year combinations were downloaded from the T3/Oat website (Saied *et al*., 2016). Data from each location and the line best linear unbiased predictor (BLUP) for heading date were used for GWAS and genomic selection comparisons. BLUP was calculated using the package lme4 implemented in R (Team R.C., 2015).

### Tag‐level haplotype and SNP analysis

The first two steps of the UNEAK pipeline were used to deconvolute and process raw reads and to produce tag‐count and merged tag‐count files (Lu *et al*., 2013). These files were then used by the Haplotag pipeline (Tinker *et al*., 2016) to call genotypes on 4657 cultivated oat lines. The following changes were made to default Haplotag parameters to accommodate the large number of taxa and/or increase stringency: as shown in the Haplotag input file (Appendix S4), the maximum number of tags in a cluster (MaxTagsToTest) was increased from the default nine to twelve. The minimum tag count (read from the merged tag‐count file) was set to 50 rather than the default value of ten. The minimum number of taxa present when selecting a model (ThreshGeno) was reduced to 0.2 from the default 0.4. The threshold for maximum heterozygote frequency (ThreshHet) was reduced to 0.08 from the default 0.1. The members of clusters with a minimum 1% minor allele frequency (MAF) were subjected to diploid segregation tests across the population. The above thresholds were used to filter a large primary data matrix, while other, more stringent thresholds were used to filter subsets of these data for further analysis, as described below.

### Marker placement on oat consensus map

Segregating Haplotag‐derived markers from ten populations (950 individuals) were used for marker placement (Appendix S2). TL haplotype and GBS‐SNP markers with a maximum of 50% missing values, >15% MAF and <10% heterozygosity were selected from the full data matrix. The genetic positions and the genotypes of the markers used for the oat consensus mapping (Chaffin *et al*., 2016) were concatenated with the new Haplotag‐derived data. The placement of new markers relative to the framework markers on the fixed consensus map was performed as described by Chaffin *et al*. (2016) and Huang *et al*. (2014). Briefly, this involved calculating the pairwise recombination rate of all the markers, placing the Haplotag‐derived markers between the two lowest recombining framework markers and interpolating the distances on the consensus map such that the original framework positions were preserved.

### Chromosome‐level haplotype analysis

CL haplotype blocks were identified in each of the full set of CORE lines (*n* = 635), the spring lines (*n* = 497) and the southern set (*n* = 123) using the method described by Gabriel *et al*. (2002) implemented in the software ‘Haploview’ (Barrett *et al*., 2005). This is an LD‐based method that computes the 95% confidence interval of pairwise marker |D’|. Marker pairs with upper bounds over 0.98 and lower bounds over 0.7 are in strong LD. However, pairs are termed ‘strong evidence for historical recombination’, if the |D’| upper bound is below 0.9. Marker pairs that do not meet either criteria are noninformative. A haplotype block is identified if 95% of the markers within a region are in strong LD. In order to meet the requirement of the software, TL haplotype markers were converted to their bi‐allelic format, which converts all minor alleles to a single alternate allele to the major allele. Block sizes in genetic distances (cM) were calculated using the genetic positions of the component markers of the haplotype blocks.

Haplotype diversity was calculated based on haplotype frequencies, $\hat{H}=\left(\frac{n}{\left(n-1\right)}\right)\left(1-\sum_{i=1}^{k}p_{i}^{2}\right)$where $\hat{H}$ = haplotype diversity, *n *= sample size, *k *= number of haplotypes in the haplotype block and *p*_*i*_= frequency of haplotypes with frequency ≥0.02 (Nei, 1987).

### Genome‐wide association mapping

Genome‐wide genotype–phenotype associations were identified using two marker systems (SNP and TL haplotype) on the same set of CORE diversity lines (*n* = 635) reported by Esvelt Klos *et al*. (2016). The TL haplotype data were converted to HapMap format using the four nucleotides plus the presence/absence codes (+/−) to recode the first six haplotypes per locus. In rare cases, where there were more than six TL haplotype at one locus, the rarest haplotypes were combined into a sixth allele code. For both TL haplotype and GBS‐SNP markers, the confounding effects of kinship (*K*) and population structure (PCA) were accounted for in the mixed linear model (MLM) implemented in TASSEL version 5 (Endelman and Jannink, 2012). Markers with MAF≥0.2 were used to calculate the centred identity by state (IBS) kinship matrix, while markers with >5% MAF and <20% missing markers were used for principal component analysis (PCA). The Bonferroni threshold with the desired ∞ = 0.05 was calculated for each marker system using the formula –log10P Bonferroni threshold = −log10 (0.05/*n*), where *n* = the number of loci. Significant GWAS hits with deflated *P* values resulting from rare (frequency<1%) haplotypes, or heterozygotes were discarded.

The CL haplotype blocks were converted to marker scores that represented the probability of the minor haplotype and imported into TASSEL. The CL haplotype blocks of the full set and their respective individual markers (TL haplotype and GBS‐SNP) were used to populate a CL haplotype incidence matrix with the dimension *i* × ((*b* × *k*)−*m*), where i is number of individuals, b is number of haplotype blocks, k is the number of alleles and m is the number of major alleles (Lorenz *et al*., 2010). Each haplotype block has (*k*−1) columns, and the haplotype incidence shows the probability that individual i carries a haplotype *k*(0,1). Individuals carrying the major haplotype 1 have 0 values in all the rows of that specific haplotype block. The R package ‘impute’ was used to impute the missing values of the incidence matrix. The incidence matrix was imported into TASSEL as a numeric marker. PCA and kinship matrix data generated using the respective TL haplotypes and GBS‐SNP markers were used for the parallel GWAS comparison of the TL haplotypes, GBS‐SNPs, CL haplotypes and a combined data set. The combined data set excluded markers that were components of the CL haplotype blocks.

### Genome‐wide scan for loci related to local adaptation

A PCA‐based genome scan for selection that is implemented in the R package pcadapt was used to identify TL haplotype markers that are correlated with population structure. TL haplotype states from the CORE diversity set (*n* = 635) with ≥5% MAF and ≤20% missing genotypes were imputed using the linkage disequilibrium‐based k‐nearest neighbour genotype imputation method, LD KNNi (Money *et al*., 2015), implemented in TASSEL. Imputed marker data were then converted to the appropriate input format for pcadapt. The first nine principal components of the CORE diversity panel were selected based on the pcadapt run with 20 principal components. Multiple regression of each marker for the selected PCA components produced the vector of *z*‐scores. The *z*‐scores were then used to calculate the Mahalanobis distance test statistic and generate *P*‐values (Duforet‐Frebourg and Slatkin, 2016; Luu *et al*., 2016a,b).The significant (∞ ≤ 0.05) outlier loci were identified after the *P*‐values were adjusted for false discovery rate or transformed to *q*‐values using the R package (*q*‐value) (Dabney *et al*., 2010).

### Genomic selection

The ridge regression best linear unbiased prediction (RR‐BLUP) algorithm implemented in the R package (rrBLUP) was used for genomic prediction (Endelman, 2011). Cross‐validation of the CORE diversity panel (*n* = 635) was conducted by taking random samples of the population as a calibration set with the remainder used as a test set. The cross‐validation to determine the optimum calibration size was performed using 13947 TL haplotype or 20373 GBS‐SNP markers with 20% maximum missing markers. The missing marker scores were imputed by the EM algorithm implemented in rrBLUP, which is the recommended method for GBS markers (Endelman, 2011; Poland *et al*., 2012). Prediction accuracy was calculated as the correlation between predicted and observed heading date values or BLUPs. As an indirect evaluation of the effect of marker density on prediction accuracy, five levels of missing values (5% to 50%) of the diversity panel were imputed, and the resulting markers were used for cross‐validation (80% calibration set and 20% test set) tests. Independent populations were subjected to genomic selection using the CORE diversity set (*n* = 635) as a calibration set and the 2010 home‐test (*n* = 197) as the test set based on 13954 TL haplotype and 20380 GBS‐SNP markers.

## Conflict of interest

The authors have no conflict of interest to declare.

## Supporting information


**Figure S1** The density of markers in the updated consensus map, based on (a) TL‐haplotype loci and (b) GBS‐SNPs.
**Figure S2** Mean chromosome haplotype diversity of the three subpopulations.
**Figure S3** Haplotype diversity of the full set of lines (a), the spring set (b), and the southern set (c) infered using the TL‐haplotype markers.
**Figure S4** The screeplot shows the proportion of explained variances against the first 20 principal components.
**Figure S5** Panel (a) shows heading date prediction accuracies of the home test 2010 (*n* = 197) population data from Prince Edward Island, calculated using the calibration data from the CORE‐diversity set (*n* = 635) from 16 locations‐years, and panel (b) shows the predicted and observed heading date values using the BLUP calibration set.
**Table S1** GWAS comparisons.
**Table S2** Outlier genomic regions in the CORE (*n* = 635).

**Appendix S1** The list of 4657 taxa genotyped and the consensus map.
**Appendix S2** The genotype data of the 950 lines used to update the consensus map (full data available by accessing haplotag.aowc.ca/SM3_OC_placement_RAW_merged.zip)
**Appendix S3** Significant GWAS hits of the different comparisons.

**Appendix S4** The input file used for the Haplotag analysis of the 4657 lines.

**Appendix S4** The input file used for the Haplotag analysis of the 4657 lines.
