# Supplementary material for: Haplotype‐based genotyping‐by‐sequencing in oat genome research
Source: Plant Biotechnol J. 2018 Mar 25;16(8):1452–63. doi: 10.1111/pbi.12888 (PMC6041447; doi:10.1111/pbi.12888)
Supplement: Supplementary file 1 — Figure S1 The density of markers in the updated consensus map, based on (a) TL‐haplotype loci and (b) GBS‐SNPs. Figure S2 Mean chromosome haplotype diversity of the three subpopulations. Figure S3 Haplotype diversity of the full set of lines (a), the spring set (b), and the southern set (c) infered using the TL‐haplotype markers. Figure S4 The screeplot shows the proportion of explained variances against the first 20 principal components. Figure S5 Panel (a) shows heading date prediction accuracies of the home test 2010 (n = 197) population data from Prince Edward Island, calculated using the calibration data from the CORE‐diversity set (n = 635) from 16 locations‐years, and panel (b) shows the predicted and observed heading date values using the BLUP calibration set. Table S1 GWAS comparisons. Table S2 Outlier genomic regions in the CORE (n = 635). [file PBI-16-1452-s003.docx]

Supplementary Tables and Figures for:

Haplotype based genotyping-by-sequencing in oat genome research

Wubishet A. Bekele, Charlene P. Wight, Shiaoman Chao, Catherine J. Howarth, and Nicholas A. Tinker

Table S1: GWAS comparisons

| GWAS set | Population | Phenotypes | Genotypes | Number of loci |
| --- | --- | --- | --- | --- |
| 1 | Diversity set (n=635) | 16 separate environments | TL-haplotypes | 12890 |
|  |  |  | GBS-SNPs | 17694 |
| 2 | Diversity set (n=635) | BLUPs across 16 environments | TL-haplotypes | 12890 |
|  |  |  | GBS-SNPs | 17694 |
|  |  |  | CL (TL-haplotypes) | 1376 |
|  |  |  | CL (GBS-SNPs) | 5100 |
|  |  |  | CL + TL-haplotypes | 12643 |
|  |  |  | CL + GBS-SNPs | 15748 |

| **Table S2:** Outlier genomic regions in the CORE (n=635) diversity panel | | |
| --- | --- | --- |
| Chrom | 1 cM bin | Frequency of significant outlier TL haplotype loci |
| 1 | 95 | 2 |
|  | 116 | 4 |
| 2 | 26 | 5 |
|  | 29 | 1 |
|  | 33 | 8 |
|  | 37 | 3 |
|  | 41 | 2 |
|  | 47 | 1 |
|  | 49 | 1 |
|  | 51 | 3 |
|  | 53 | 2 |
|  | 54 | 2 |
|  | 59 | 1 |
|  | 62 | 2 |
|  | 66 | 13 |
|  | 67 | 1 |
|  | 70 | 15 |
|  | 71 | 11 |
|  | 72 | 61 |
|  | 78 | 2 |
|  | 81 | 10 |
|  | 82 | 1 |
|  | 84 | 66 |
|  | 85 | 8 |
|  | 86 | 120 |
|  | 96 | 1 |
|  | 97 | 2 |
|  | 98 | 2 |
|  | 99 | 1 |
|  | 105 | 2 |
|  | 107 | 14 |
| 3 | 104 | 1 |
|  | 161 | 11 |
| 5 | 124 | 1 |
| 6 | 137 | 2 |
| 8 | 148 | 1 |
|  | 152 | 4 |
| 11 | 45 | 4 |
|  | 48 | 6 |
|  | 50 | 1 |
|  | 52 | 157 |
|  | 53 | 20 |
|  | 55 | 1 |
|  | 56 | 1 |
|  | 57 | 1 |
|  | 67 | 4 |
|  | 68 | 7 |
|  | 69 | 1 |
|  | 100 | 1 |
|  | 101 | 1 |
|  | 103 | 1 |
| 12 | 44 | 1 |
| 15 | 30 | 3 |
|  | 31 | 6 |
|  | 32 | 75 |
|  | 33 | 48 |
|  | 34 | 153 |
|  | 85 | 1 |
| 17 | 23 | 1 |
|  | 25 | 3 |
|  | 29 | 2 |
|  | 31 | 4 |
|  | 32 | 86 |
|  | 33 | 79 |
|  | 35 | 6 |
|  | 36 | 7 |
|  | 109 | 14 |
| 18 | 36 | 45 |
|  | 39 | 2 |
|  | 40 | 3 |
|  | 41 | 4 |
|  | 42 | 2 |
|  | 58 | 1 |
|  | 59 | 9 |
|  | 61 | 15 |
|  | 62 | 4 |
|  | 63 | 4 |
|  | 64 | 1 |
|  | 67 | 37 |
|  | 68 | 37 |
|  | 79 | 2 |
|  | 80 | 4 |
|  | 81 | 2 |
| 20 | 71 | 1 |
|  | 96 | 1 |
|  | 119 | 1 |
|  | 122 | 4 |
|  | 143 | 4 |
| 28 | 19 | 3 |
|  | 23 | 1 |
|  | 32 | 9 |
|  | 35 | 8 |
|  | 36 | 65 |
|  | 38 | 3 |
|  | 39 | 17 |
|  | 40 | 1 |
|  | 43 | 238 |


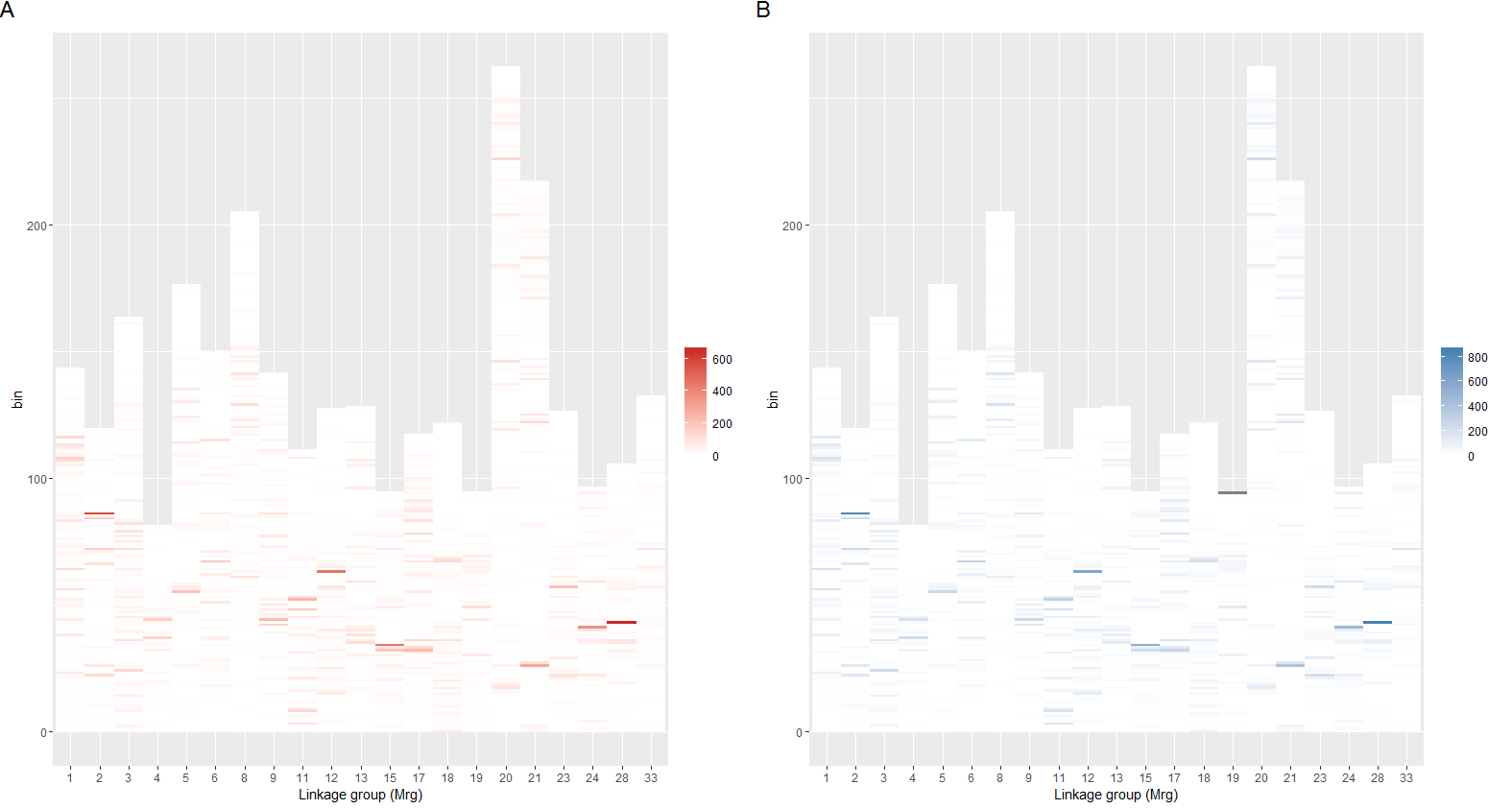


Figure S1. The density of markers in the updated consensus map, based on (A) TL-haplotype loci and (B) GBS-SNPs. The horizontal axis shows the chromosome representations (linkage groups), the vertical axis shows the 1cM bins, and the colour intensity shows the number of markers per bin.


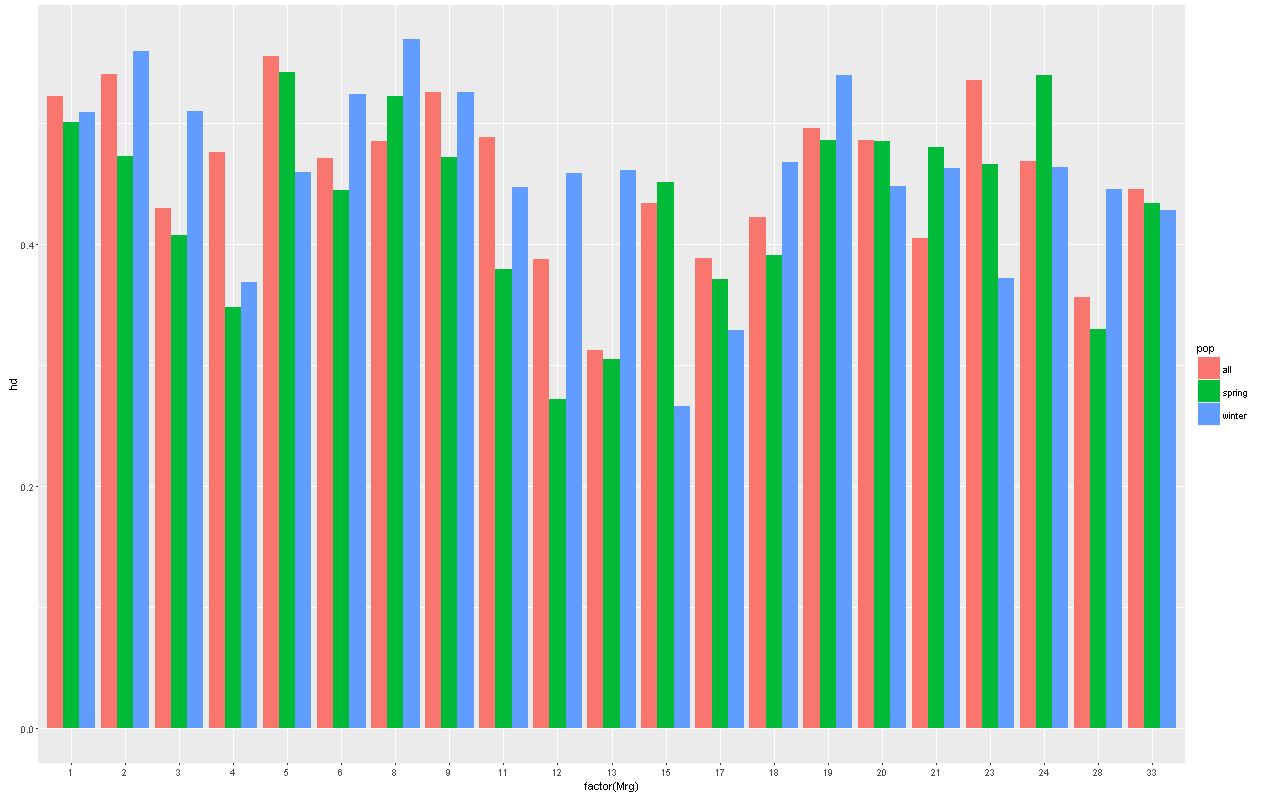


Figure S2: Mean chromosome haplotype diversity of the three subpopulations.


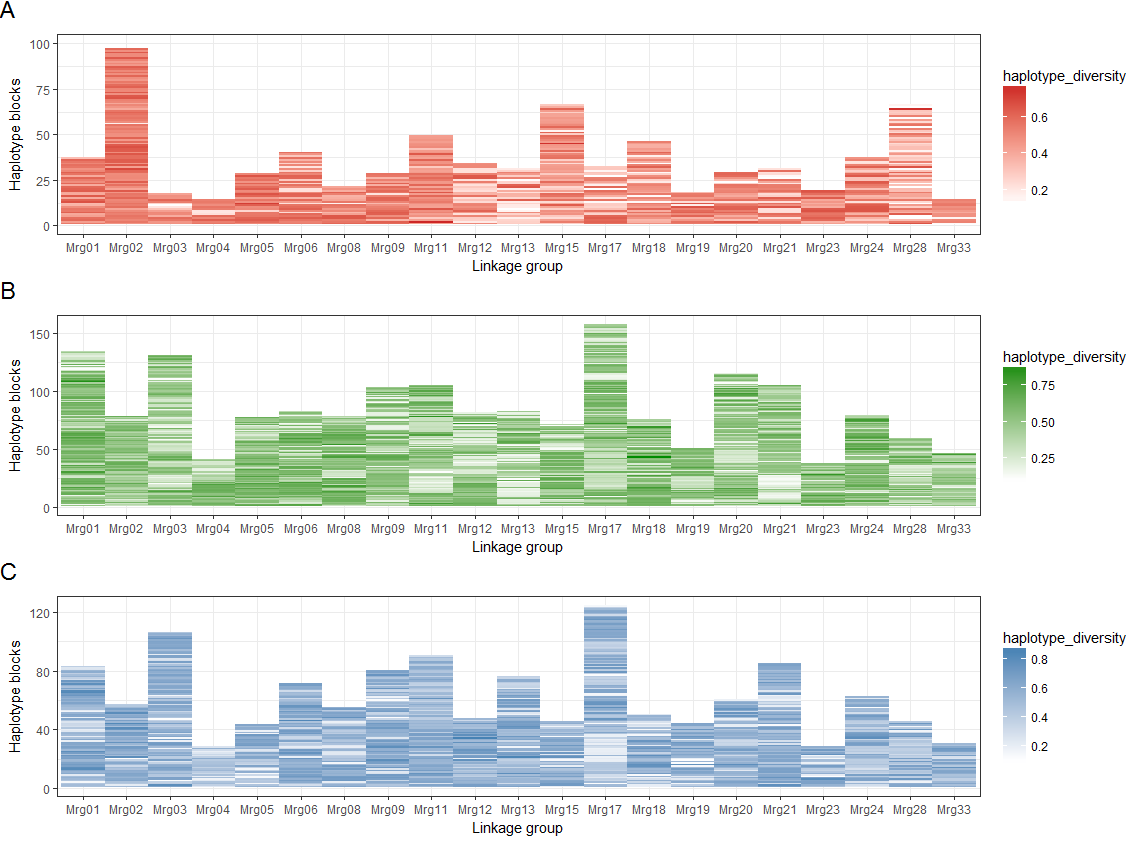


Figure S3: Haplotype diversity of the full set of lines (A), the spring set (B), and the southern set (C) infered using the TL-haplotype markers. The horizontal axis shows the 21 chromosome representations from the oat consensus map.. Each solid bar indicates a haplotype block, stacked in sequential map order, such that the vertical scale represents the number of haplotype blocks on each chromosome.The haplotype diversity within each haplotype block is indicated by colour intensity.


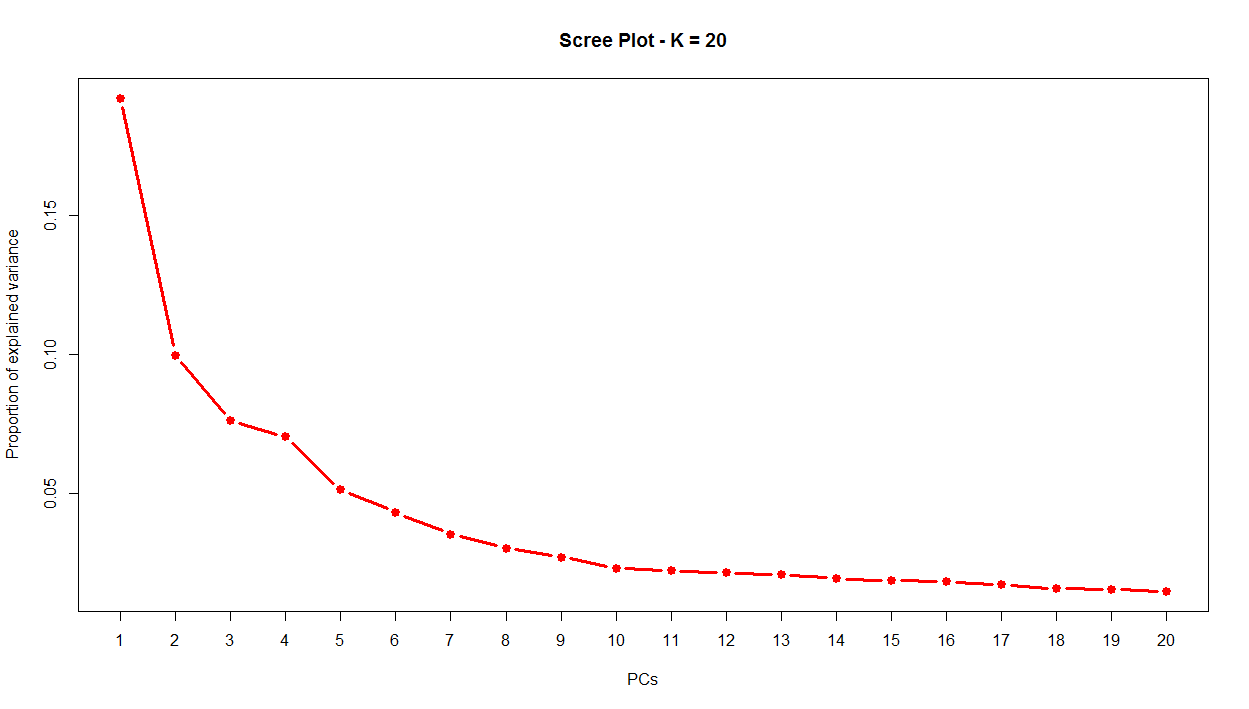


Figure S4: The screeplot shows the proportion of explained variances against the first 20 principal components.


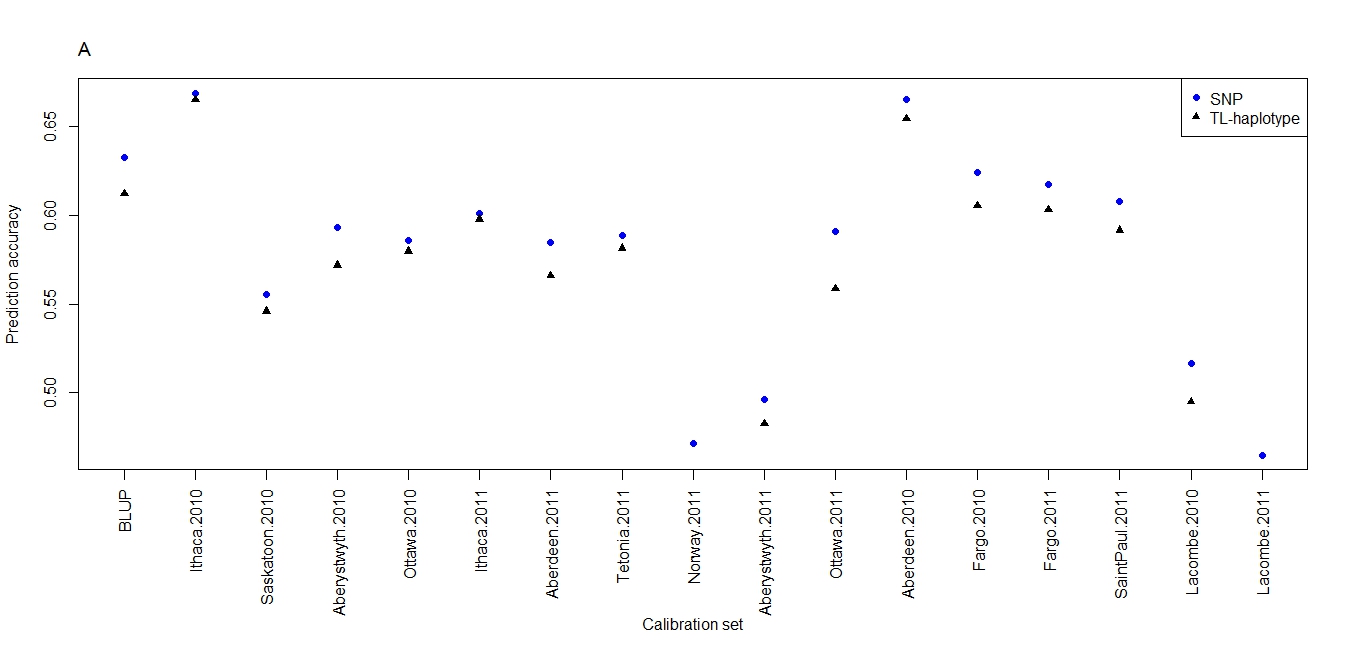


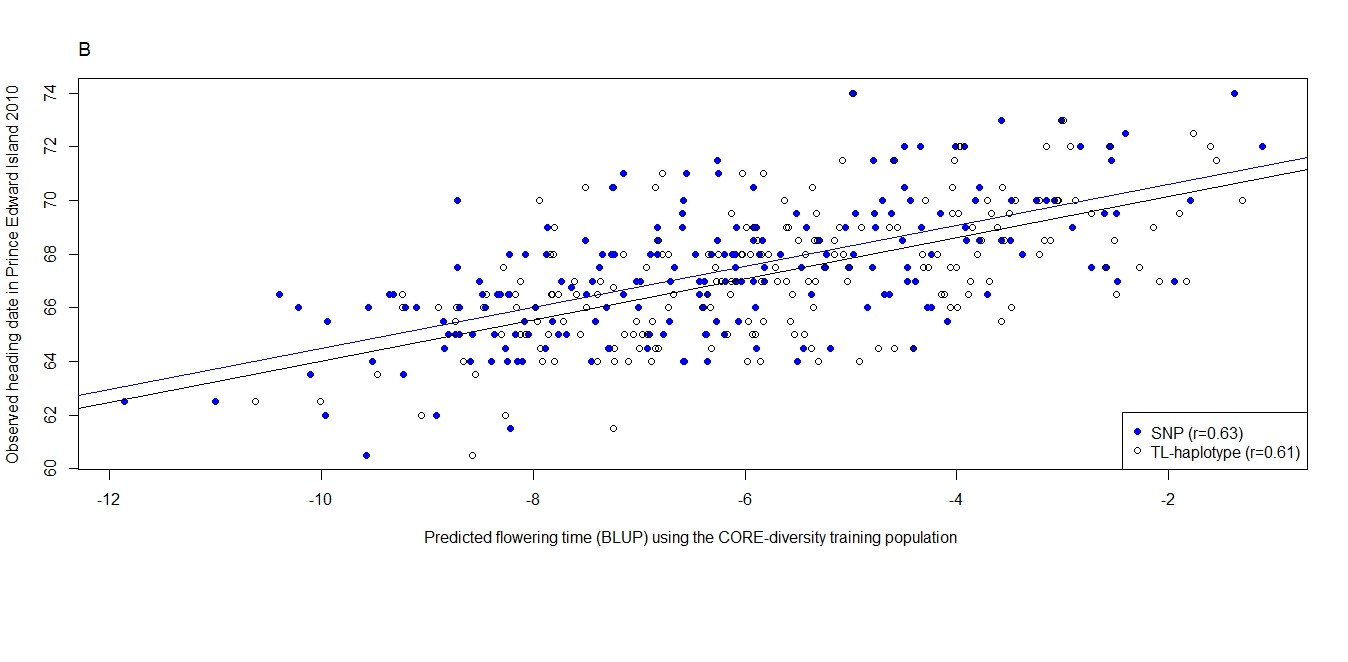
Figure S5: Panel (A) shows heading date prediction accuracies of the home test 2010 (n=197) population data from Prince Edward Island, calculated using the calibration data from the CORE-diversity set (n=635) from 16 locations-years, and panel (B) shows the predicted and observed heading date values using the BLUP calibration set.
